# Effect of the local wind reduction zone on seed dispersal from a single shrub element on sparsely vegetated land

**DOI:** 10.1093/aobpla/plab025

**Published:** 2021-05-21

**Authors:** Lin-Tao Fu

**Affiliations:** School of Architecture and Civil Engineering, Chengdu University, Chengdu 610106, Sichuan, China

**Keywords:** Dispersal kernel, long-distance dispersal, release height, vegetation porosity, wind intensity

## Abstract

Accurate predictions of seed dispersal kernels are crucial for understanding both vegetation communities and landscape dynamics. The influences of many factors, including the physical properties of seeds, the time-averaged wind speed and the wind turbulence, on seed dispersal have been studied. However, the influence of local wind speed reduction around a single shrub element (e.g. a small patch of scrub) on seed dispersal is still not well understood. Here, the spatial distribution of the wind intensity (represented by the wind friction speed u_*_) around a single shrub element is described, with an emphasis on the variation in the streamwise direction, and assuming that the time-averaged lateral and vertical speeds are equal to zero. The trajectories of the seeds were numerically simulated using a Lagrangian stochastic model that includes the effects of wind turbulence and particle inertia. The patterns of seed deposition with and without the effect of local wind reduction were compared. The variation in seed deposition with changing wind intensity, release height and shrub porosity were also simulated. The simulation results revealed that the local wind reduction increased seed deposition in nearby regions and therefore decreased seed deposition in the regions farther away. Local wind reduction had a greater impact on short-distance dispersal than on long-distance dispersal. Moreover, the dispersal in the circumferential direction decreased once the motion of a seed moving in the streamwise direction was reduced due to the local wind reduction. As the wind intensity and release height increased, the effect of local wind reduction on seed dispersal weakened. Seed dispersal was both wider and farther as the shrub porosity increased. These results may help explain the disagreement between the mechanistic models and the fitting curves in real cases. In addition, the results of this study may improve the currently used mechanistic models by either increasing their flexibility in case studies or by helping explain the variations in the observed distributions.

## Teaser

In this study the trajectories of the seeds released from a single shrub element were numerically simulated through considering the spatial distribution of the wind intensity around the shrub element. The simulation results revealed that local wind reduction increased seed deposition in nearby regions and therefore decreased seed deposition in the regions farther away. Wind intensity and release height weaken the effect of local wind reduction. Shrub porosity promotes seed dispersal by wind. These results may help explain the disagreement between the mechanistic models and the fitting curves in real cases.

## Introduction

Wind-driven seed dispersal is important in both microscale and macroscale ecological systems (e.g. Howe and Smallwood 1982; Nathan *et al.* 2011; Beckman *et al.* 2020a). The dynamics of ecosystems can only be reasonably modelled or predicted when accurate information regarding seed dispersal kernels (i.e. the probability distribution functions of the dispersed seeds) is available (Beckman *et al.* 2020b). Therefore, many studies, including field observations (e.g. Dorp *et al.* 1996; Bullock and Clarke 2000; Nathan *et al.* 2002b), theoretical derivations (e.g. Greene and Johnson 1989; Katul *et al.* 2005) and numerical simulations (e.g. Schippers and Jongejans 2005; Duman *et al.* 2016), have been conducted to obtain the distribution functions of dispersed seeds.

A variety of functions, such as exponential, power, log-sech, inverse Gaussian and Weibull functions, have been proposed for dispersal kernels based on their specific environmental settings (Heydel *et al.* 2014; Bullock *et al.* 2017, 2018; Cousens *et al.* 2018). On the one hand, owing to the complexity of real cases, theoretical derivations cannot always reproduce field observations because many of the parameters have been simplified. On the other hand, field measurements cannot both gather all of the dispersed seeds from the plants and provide sufficient wind information, which suggests that empirical functions are usually case dependent.

Compared to both theoretical derivations and field observations, numerical simulation is a more economical and flexible method of studying the dispersal of seeds by wind in both ideal cases and complicated field cases (Bullock and Clarke 2000; Schippers and Jongejans 2005; Kuparinen *et al.* 2007; Bohrer *et al.* 2008; Duman *et al.* 2016). However, the reliability of simulation results depends on having a comprehensive and accurate description of the factors that can affect the dispersal of seeds. Typically, once a seed is released, the influencing factors can be sorted into two types. One type originates from the seed itself. For example, seed size, density and morphology can each affect the aerodynamic drag coefficient and the vertical terminal settling velocity of the seeds (Greene and Quesada 2005). The second type is the driving vector—wind speed. In earlier studies, only the time-averaged wind speed was used to estimate the dispersal kernel (e.g. Greene and Johnson 1989; Nathan *et al.* 2001).

Later, turbulence was found to be crucial to seed wind dispersal, especially for long-distance dispersal (e.g. Katul *et al.* 2005). The influence of the vegetation density, which is parameterized using either the canopy attenuation coefficient or the leaf area index, has also been included to help describe the wind and turbulence in the presence of multiple plants (Raupach *et al.* 1991; Kaimal and Finnigan 1994). Thus, seed dispersal kernels can vary with vegetation density (Nathan *et al.* 2002a). Geographical features such as hills can also affect both wind and turbulence, and consequently seed dispersal (Trakhtenbrot *et al.* 2014). Recently, it was found that the intermittency of the turbulence dissipation rate increased the long-distance dispersal of seeds in a forest landscape (Duman *et al.* 2016).

Generally, in previous studies, wind speed has usually been assumed to be only height-dependent, and variation in wind speed in the streamwise direction has rarely been considered. Although the effect of wind reduction on seed dispersal when considering the distance from the edge of the forest to a clearing has been recognized (Greene and Johnson 1995, 1996), the effect of the wind reduction region, which is formed both within and in the lee of a single vegetation element in an open landscape, on seed dispersal has not been studied. For a single taller tree, the local wind reduction may have a limited impact on seed dispersal (Greene and Johnson 1996). Nevertheless, for a single vegetation element with a low height, the effect of the local wind reduction on seed dispersal may be significant because of the low wind speed at a low height in the atmospheric boundary layer. Once a seed is released, it may immediately move into the wind reduction region. Thus, the reduction region could thus dominate the early stage of seed dispersal process, which may be very important for the subsequent trajectories of seeds.

Measurements and simulations (e.g. Leenders *et al.* 2007; Yang *et al.* 2016; Liu *et al.* 2018) have revealed that the lateral and leeward flow around vegetation is affected by the presence of vegetation. In other words, the interaction between the vegetation and air flow changes the spatial distribution of the wind speed not only in the vertical direction but also around the vegetation element as well. Furthermore, the distribution of the wind reduction regions is not only affected by the vegetation height but also affected by the porosity of the vegetation elements (Leenders *et al.* 2011; Mayaud *et al.* 2017). The porosity is one parameter that can be used to describe the density level of a single vegetation element, and it is typically represented by the optical porosity (ranging from 0 to 1). A porosity value of 0 indicates a solid element with a strong effect on air flow, while a porosity value of 1 indicates a fully porous structure with no effect on air flow.

Shrubs, which reduce the risk of wind erosion, are an important vegetation type in several places, such as in arid regions and the edges of deserts (Okin 2008; Mayaud *et al.* 2017). Therefore, it is important to predict the development of a shrub community through seed dispersal. In this study, the influences of the local wind reduction on seed dispersal around a single shrub element were investigated, with emphasis on the local wind reduction in the streamwise direction. The spatial distribution of the local wind reduction and its parameterization are described in detail. All of the seeds were assumed to be released from a point source from a single shrub element standing alone on a surface with a low roughness. In addition to the traditional probability distributions of the deposited seeds in the radial directions, the deviation of the dispersal distance in the circumferential direction (hereafter called ‘circumferential displacement’) was also investigated, because of the heterogeneous variations of the streamwise wind speed reduction in the lateral direction. The variations in the effects of the local wind reduction on seed dispersal were investigated, and the wind intensity, release height and vegetation porosity were taken into account.

## Materials and Methods

**Figure 1.**
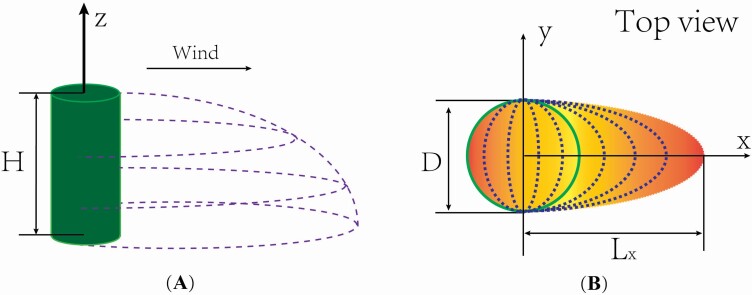
Illustrations of the local wind reduction in the lee of a single shrub element (A) and the distribution pattern of the wind friction velocity near the surface both within and in the lee of the shrub element (B). *H* and *D* denote the shrub height and diameter, respectively, and *L*_*x*_ denotes the maximum streamwise length of the wind reduction region. (A) The region enclosed by dashed lines is the wind reduction region. (B) The colour depth along the streamwise direction suggests the change of the wind friction velocity (light colour denotes low velocity); the dotted lines suggest the identical friction velocities.

Owing to the diversity of shrub shapes in nature, for convenience, the shape of a single vegetation element was assumed to be a circular cylinder (Fig. 1A), as per previous studies (Raupach 1992; Okin 2008). The goal of this study was to investigate seed dispersal over a sparsely vegetated land surface, meaning that interactions among neighbouring shrub elements could be ignored (Leenders *et al.* 2007; Mayaud *et al.* 2017; Fu *et al.* 2019). A surface with a low roughness was used, based on the assumption that the ground surface was not very rough and that the height of the ground vegetation cover was small. The origin of the Cartesian coordinate system was fixed at the centre of the circular cylinder base. All of the seeds were assumed to be perfectly spherical.

### Wind spatial distribution including local wind speed reduction

In real situations, the air movement around a single vegetation element is very complex due to the coupling responses between the vegetation and air flow (Mayaud and Webb 2017; Kang *et al.* 2019). Many studies have been conducted on the air around cylindrical elements (e.g. Kawamura *et al.* 1984; Rostamy *et al.* 2012; Fu *et al.* 2019). These studies have found that a local wind reduction region can form in the lee of a circular cylindrical element. The flow patterns (e.g. the vortex structures) in this region are diverse and depend on the boundary thickness, surface roughness, cylinder height and Reynolds number. However, in this study, the average lateral wind speed and average vertical wind speed were assumed to be zero for three reasons. First, the average vertical wind speed in the leeward wind reduction region of a solid circular cylinder with a low area ratio (frontal area vs. basal area) is low (Rostamy *et al.* 2012). Second, the vegetation porosity could affect both the vertical and lateral wind speeds (Lv *et al.* 2014; Taddei *et al.* 2016; Tang *et al.* 2019). Third, the seed release point was positioned in the centre of the shrub element, meaning that the effects of both the lateral and vertical winds on the seeds’ trajectories in a wind reduction region could thus be limited. In fact, detailed field measurements of the lateral and vertical wind speeds around a shrub element are rare in comparison to streamwise wind speed measurements. Therefore, this study mainly focuses on the effects of streamwise wind reduction on seed dispersal.

The wind speed at any location over a flat surface could be expressed by Equation (1) (Raupach 1992):


$$\begin{array}{l} U(x,y,z)=\frac{u_{*}(x,y,z)}{\kappa }ln\left(\frac{z}{z_{0}}\right) \end{array}$$ (1)

where *U*(*x*, *y*, *z*) and u_*_(*x*, *y*, *z*) are the time-averaged streamwise speed and wind friction speed, respectively. The *x*, *y* and *z* coordinates correspond to the streamwise, lateral and vertical directions, respectively. κ is the von Karman’s constant and is usually taken as 0.41. In all of the calculations, the aerodynamic surface roughness *z*_0_ was assumed to be constant. The description of the wind speed distribution in this work was divided into two parts in accordance with previous studies (e.g. Raupach 1992; Okin 2008; Leenders *et al.* 2011), and as shown in Fig. 1. The first part includes vegetation elements and the leeward wind speed reduction region (Fig. 1A). The second part is the remaining region, in which the flow is not disturbed by the vegetation element (or the disturbance is so weak that it could be ignored). For the undisturbed region, the friction speed was set to be equal to the friction speed of the incoming flow, u_*_, as was done in previous studies (Bullock and Clarke 2000; Nathan *et al.* 2002a). The determination of the wind speed in the first part is relatively complicated because the friction speed of the streamwise wind was assumed to be spatially variable.

First, the friction speed at ground level along the streamwise direction should be defined. Triangles, rectangles and half-ellipses have been proposed for the basal shape of the wind reduction region in the lee of plants (Raupach 1992; Okin 2008; Leenders *et al.* 2011). Recent observations (Leenders *et al.* 2011; Chen *et al.* 2012; Mayaud *et al.* 2017) and simulations (Yang *et al.* 2016; Sadique *et al.* 2017) have indicated that the half-ellipse scheme proposed by Leenders *et al.* (2011) seems to be more reasonable for porosity vegetation elements, as is shown in Fig. 1B. The semi-minor axis of the half-ellipse used here was set to be *D*/2 (where *D* is the diameter of the circular cylinder) (Leenders *et al.* 2011). The maximum streamwise length (*L*_*x*_) of the wind reduction region (i.e. the semi-major axis) is set as 7.5*H* (where *H* is the height of the circular cylinder) (Leenders *et al.* 2011).

The half-ellipse scheme suggests that the streamwise wind reduction in the lateral direction is heterogeneous. This indicates that, for a fixed value of *x* (i.e. the streamwise location coordinate), the friction speeds at different values of *y* over the ground (*z* = 0) within the wind reduction region are different. For example, Leenders *et al.* (2011) assumed that the friction speed increases exponentially with increasing the leeward distance from the leeward edge of a shrub element towards the maximum streamwise length in the form of a half-ellipse contour (Fig. 1B). According to another study (Chen *et al.* 2012), the ground friction speed within a porous shrub element was assumed to decrease linearly from the windward edge of the element to the leeward edge, also in the form of half-ellipse contour (Fig. 1B). Thus, the spatial distribution of the friction speed on the ground $u_{*}(x,y,0)$ within the wind reduction region was determined through two steps. The first step was using Equations (2a) and (2b) (Leenders *et al.* 2011; Chen *et al.* 2012) to find the friction speed at the location where *x* = *L*_*x*0_ (*L*_*x*0_ is the streamwise distance to the origin of the coordinate system) along the central line (i.e. along the line *y* = 0 in plane *z* = 0; **see****Supporting Information—Fig. S1**). The second step was to assign $u_{*}(L_{x0},0,0)$ to $u_{*}(x,y,0)$ when *x* and *y* satisfy the ellipse equation ${\left(\frac{x}{L_{x0}}\right)}^{2}+{\left(\frac{y}{0.5D}\right)}^{2}=1$ (the dotted lines in Fig. 1B). Here, according to Mayaud *et al.* (2017), u_*0_ = (1.46 × *θ* − 0.4076)u_*_, and *b* = 1.05 × *θ* + 0.1627, where u_*0_ is the minimum friction speed at the leeward edge of the shrub element, *θ* is the shrub porosity and *b* is the speed recovery rate of a shrub element of height *H*.


$$u_{*}\;(L_{x0},0,0)=(u_{*}+u_{*0})/2-(u_{*}-u_{*0})L_{x0}/D\;if\;-D/2\leq L_{x0}\leq D/2$$ (2a)

$$u_{*}\;(L_{x0},0,0)=(u_{*}-u_{*0})[1-exp(-L_{x0}b/H)]+u_{*0}\;if\;D/2<L_{x0}\leq L_{x}$$ (2b)

Next, the vertical variation in the friction speed within a wind reduction region was calculated. Previous studies (Rostamy *et al.* 2012; Lv *et al.* 2014; Taddei *et al.* 2016) have suggested that the spatial morphology of the wind reduction region in the lee of a single shrub element could be roughly depicted by a quarter of an ellipsoid (Fig. 1A). Thus, the spatial distribution of the friction speed at height *z* (in the *z* plane within the wind reduction region) could be expressed by the half-ellipse scheme mentioned above, with a height-dependent half-ellipse ${\left(\frac{x}{L_{xz}}\right)}^{2}+{\left(\frac{y}{0.5D}\right)}^{2}=1$ (0.5*D* < *L*_*xz*_ < *L*_*x*_), where *L*_*xz*_ is the maximum streamwise length of the wind reduction region at height *z*. Moreover, the continuity of the wind speed at the interface between the wind reduction region and the undisturbed region should be maintained. Here, for simplicity, the value of the friction speed at a location (*x*, *y*, *z*) was assumed to linearly increase from the ground to the interface as the vertical coordinate *z* increased (Equation (2c)):


$$u_{*}\;(x,y,z)=u_{*}(x,y,0)+z(u_{*0}-u_{*}(x,y,0))/z_{hxy}\;if\;0\leq z\leq z_{hxy}$$ (2c)

where *z*_*hxy*_ is the height (i.e. the distance from the ground to the interface) of the wind reduction region at location (*x*, *y*). Note that the height of local wind reduction region is limited by shrub height (Fig. 1A), which suggests $0\leq z_{hxy}\leq H$.

The influence of the atmospheric turbulence on seed movement was also considered. Thus, the instantaneous wind speed consists of two parts: a time-averaged wind speed and a turbulence fluctuation speed. Therefore, the instantaneous wind speeds in three directions could be written as $u=U+u^{\prime }$, $v=V+v^{\prime }$ and $w=W+w^{\prime }$. The prime represents a speed fluctuation. The streamwise speed *U* was determined using Equation (1). Although the time-averaged lateral speed *V* and vertical speed *W* were assumed to be zero, the effects of speed fluctuations in these two directions on seed movements were still taken into account. Because empirical wind speed and turbulence parameterizations were used and heavy particles are likely to violate the well-mixed conditions by accumulating in low turbulence areas (Arritt *et al.* 2007), the well-mixed condition assumption (e.g. Thomson 1987; Wilson and Sawford 1996; Wilson 2000; Duman *et al.* 2016) was not applied here. Instead, the variations in the turbulence fluctuations along the trajectory were statistically described using Equations (3a)–(3c), as in previous studies (Van Dop *et al.* 1985; Arritt *et al.* 2007; Kok and Renno 2009):

$$\begin{array}{l} u^{\prime }(t+dt)-u^{\prime }(t)=-u^{\prime }(t)dt/T_{l}+n_{Gu}\sigma_{u}\sqrt{2dt/T_{l}} \end{array}$$ (3a)

$$\begin{array}{l} v^{\prime }(t+dt)-v^{\prime }(t)=-v^{\prime }(t)dt/T_{l}+n_{Gv}\sigma_{v}\sqrt{2dt/T_{l}} \end{array}$$ (3b)

$$\begin{array}{l} w^{\prime }(t+dt)-w^{\prime }(t)=-w^{\prime }(t)dt/T_{l}+n_{Gw}\sigma_{w}\sqrt{2dt/T_{l}} \end{array}$$ (3c)

where *n*_G_ is a Gaussian distributed random number with zero mean and unit standard deviation (SD). $\sigma_{u}$, $\sigma_{v}$ and $\sigma_{w}$ are the SDs of the wind fluctuations. Based on previous studies (Nishimura and Hunt 2000), $\sigma_{u}$ = 2.5u_*_(*x*, *y*, *z*), and $\sigma_{v}$ = $\sigma_{w}$ = 1.3u_*_(*x*, *y*, *z*). The Lagrangian timescale and the time-averaged turbulent dissipation rate were defined as *T*_l_ = 2$\sigma_{w}^{3}$/(*C*_0_$\varepsilon_{0}$) and $\varepsilon_{0}$ = u_*_(*x*, *y*, *z*)^3^/κ*z*, respectively. *C*_0_ was set as 4.0 (Jarosz *et al.* 2004; Duman *et al.* 2016). Equation (3) suggests that the dissipation intermittency was not included here.

### Master equation of seed motion

Although the motion of seeds in the atmospheric boundary layer is subject to many forces (Maxey and Riley 1983), for simplicity, only gravity and drag were considered in this study. In addition, the translational movement of the seeds was calculated while the rotation was ignored, in accordance with the study of Duman *et al.* (2016). For a seed with an average diameter of *d*_s_, its mass *m* is equal to $\pi \rho_{s}d_{s}^{3}/6$, where $\rho_{s}$ is the seed density. The drag force acting on the seed can be estimated as $F_{drag}=\frac{\pi }{8}\rho_{a}d_{s}^{2}C_{d}\left|\vec{V_{r}}\right|\vec{V_{r}}$ (Anderson 1987), where $\rho_{a}$ is the air density, *C*_D_ is the drag coefficient, which is defined as *C*_D_ = [(32/Re_D_)^2/3^ + 1]^3/2^ for irregular particles (Cheng 1997). $Re_{D}=\rho_{a}d_{s}\left|\vec{V_{r}}\right|/\mu$, where μ is the dynamic viscosity of air, and $\vec{V_{r}}$ is the speed of the particles relative to the surrounding flow. Thus, the seed’s motion in three directions could be calculated using Equation (4) when turbulence is included:


$$\begin{array}{ll} \frac{d^{2}x_{p}}{dt^{2}} & =\frac{F_{drag,x}}{m}=-\frac{3}{4}\frac{\rho_{a}C_{D}}{\rho_{s}d_{s}}\times \left(\frac{dx_{p}}{dt}-u\right)\\ & \times \sqrt{{\left(\frac{dx_{p}}{dt}-u\right)}^{2}+{\left(\frac{dy_{p}}{dt}-v\right)}^{2}+{\left(\frac{dz_{p}}{dt}-w\right)}^{2}} \end{array}$$ (4a)

$$\begin{array}{ll} \frac{d^{2}y_{p}}{dt^{2}}= & \frac{F_{drag,y}}{m}=-\frac{3}{4}\frac{\rho_{a}C_{D}}{\rho_{s}d_{s}}\times \left(\frac{dy_{p}}{dt}-v\right)\\ & \times \sqrt{{\left(\frac{dx_{p}}{dt}-u\right)}^{2}+{\left(\frac{dy_{p}}{dt}-v\right)}^{2}+{\left(\frac{dz_{p}}{dt}-w\right)}^{2}} \end{array}$$ (4b)

$$\begin{array}{ll} \frac{d^{2}z_{p}}{dt^{2}}= & \frac{F_{drag,z}}{m}=-\frac{3}{4}\frac{\rho_{a}C_{D}}{\rho_{s}d_{s}}\times \left(\frac{dz_{p}}{dt}-w\right)\\ & \times \sqrt{{\left(\frac{dx_{p}}{dt}-u\right)}^{2}+{\left(\frac{dy_{p}}{dt}-v\right)}^{2}+{\left(\frac{dz_{p}}{dt}-w\right)}^{2}}-g \end{array}$$ (4c)

where *x*_*p*_, *y*_*p*_ and *z*_*p*_ represent the locations of the seeds in the horizontal, lateral and vertical directions, respectively, and *g* is the gravitational acceleration.

### Other settings

Based on previous studies, the constants used to calculate the seed trajectories are presented in Table 1. The initial speeds of the released seeds in the three directions were set to zero. The seed trajectories were numerically predicted using the fourth-order Runge–Kutta method (Hildebrand 1956). Because of the trajectory-crossing effect caused by inertia and gravity, the Lagrangian timescale was adjusted (Csanady 1963; Arritt *et al.* 2007). In contrast to previous investigations (Wilson 2000; Arritt *et al.* 2007) in which the terminal settling velocity was used, the relative speed between the particle and the flow was adopted to depict the trajectory-crossing effect (Anderson 1987). Thus, the modified Lagrangian time scale ($T_{pz}$) is estimated using Equation (5):

**Table 1.** Some constants used in this work. *D*, *H/D*, *d*_s_, $\rho_{s}$, $\rho_{a}$, μ, *g* and *z*_0_ are shrub diameter, ratio of shrub height to diameter, seed diameter, seed density, air density, dynamic viscosity of air, gravitational acceleration and aerodynamic surface roughness, respectively.

*D* (m)	*H/D*	*d*_s_ (mm)	*ρ*_s_ (kg m^−3^)	*ρ*_a_ (kg m^−3^)	*μ* (kg m^−1^ s^−1^)	*g* (m s^−2^)	*z*_0_ (m)
1^a^	0.5^a^	0.5^b^	500^b^	1.225	1.78 × 10^−5^	9.81	0.001^c^

^a^Based on Bullock and Clarke (2000), Leenders *et al.* (2011) and Mayaud *et al.* (2017).

^b^Based on Bullock and Clarke (2000) and Venable *et al.* (2008).

^c^Based on Raupach *et al.* (1991).


$$\begin{array}{l} T_{pz}=\frac{T_{l}}{\sqrt{1+{\left(\beta V_{r}/\sigma_{w}\right)}^{2}}} \end{array}$$ (5)

where β = 1.0 (Kok and Renno 2009). Thus, the discrete time step for the trajectory calculation according to the Runge–Kutta method was determined to be *dt* = 0.01 × *T*_*pz*_.

The inclusion of the turbulence effect (Equation (3)) leads to variations in the trajectories of seeds with the same initial speed and wind intensity conditions. Therefore, 10 stochastic runs (10^6^ seeds were released in a single run) were performed for each defined calculation condition. Owing to the variable wind speed, all of the results described below are the average values for 10 runs. To obtain the statistical information for seed dispersal kernels, starting at the central location of the shrub element, 100 discrete grids were applied along the streamwise direction. The intervals of the 1st–10th, 11th–20th, 21th–30th, 31th–40th and 41th–50th grids are 0.1*H*, 0.2*H*, 0.3*H*, 0.4*H* and 0.5*H*, respectively. The intervals of the remaining grids are 1*H*. Then, the distribution function and cumulative probability of the dispersed seeds were calculated and analysed grid by grid. Two streamwise distances, i.e. the L50 for which the cumulative probability reaches 50 % and the L99 for which the cumulative probability reaches 99 %, were recorded to help with the data analyses. Furthermore, the circumferential displacement (dc) (**see****Supporting Information—Fig. S2** for the definition of the circumferential displacement) caused by the lateral turbulence was also recorded. The average circumferential displacement ($\bar{dc}$) and the SD of the circumferential displacement ($\sigma_{dc}$) were calculated as $\bar{dc}=\left(\sum_{i=1}^{i=n}\left|dc_{i}\right|\right)/n\;$ and $\sigma_{dc}=\sqrt{\sum_{i=1}^{i=n}{\left(\left|dc_{i}\right|-\bar{dc}\right)}^{2}/(n-1)}$ in each grid, respectively, where *n* is the total number of seeds in a grid. To eliminate the high scatter caused by the generation of random numbers in the grids where deposited seeds are very rare, the statistics for both $\bar{dc}$ and $\sigma_{dc}$ started from the grid in which the cumulative probability of the deposited seeds reached 1 %, and ended by the grid in which the cumulative probability reached 99 %. Moreover, to clarify the role of shrub porosity, the L50, L99, maximum $\bar{dc}$ and normalized maximum $\sigma_{dc}$ among all grids were normalized using the values for a shrub porosity θ = 1.0 (i.e. without considering the local wind reduction).

The lowest friction speed that could cause seed release was assumed to be 0.2 m s^−1^ (Schippers and Jongejans 2005). To investigate how wind intensity affects the influence of the local wind reduction on seed dispersal, five sets of uniform wind intensity values (u_*_ = 0.2, 0.3, 0.4, 0.5 and 0.6 m s^−1^) were used for the incoming air flow. Additionally, five seed release heights (*H*_r_ = 1.0*H*, 0.9*H*, 0.7*H*, 0.5*H* and 0.3*H*) were used. When the effect of the local wind reduction was not considered, the shrub porosity was equal to 1. The other five values of porosity, which were based on recent field observations, were 0.3, 0.4, 0.5, 0.6 and 0.7 (Mayaud *et al.* 2017). Under natural conditions, wind intensity is not temporally uniform, but rather it follows the Weibull distribution law. For the hourly-averaged wind speed, the cumulative probability of the friction speed is expressed as $f(u_{*};k,c)=1-exp[-{\left(u_{*}/c\right)}^{k}]$, where *k* = 2 (Seguro and Lambert 2000), and $c=2u_{*a}/\sqrt{\pi }$. u_*a_ is the average friction speed, where the subscript ‘a’ indicates an average value. Here, to compare the seed deposition pattern that includes the local wind reduction with the pattern that does not include it, the Weibull distribution of the wind intensity was used for the incoming air flow, where u_*a_ = 0.30 m s^−1^ with a maximum friction speed cut-off of u_*_ = 0.65 m s^−1^.

### Verification of the model

**Figure 2.**
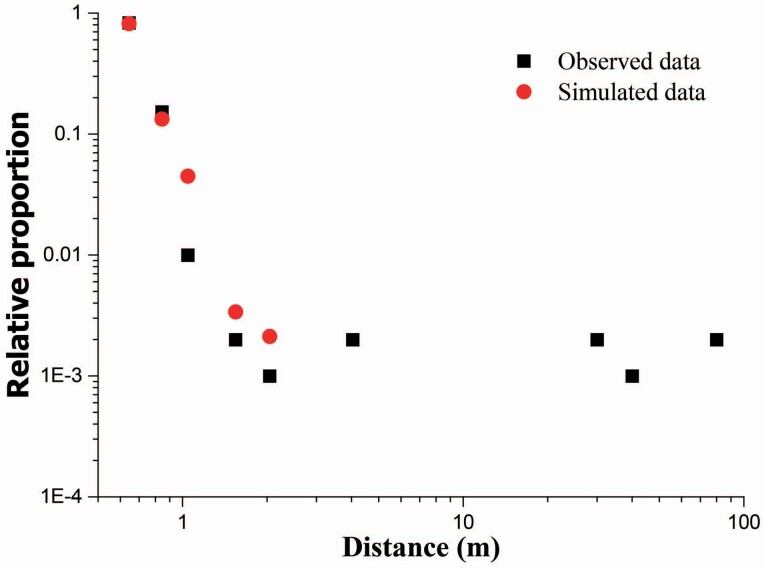
The variations of the relative proportion along with the leeward distance. Solid square symbols and solid circle symbols denote the observed data and the simulated data, respectively.

The numerical model was verified using the observed data for heather *Calluna vulgaris* presented in Table 3 of Bullock and Clarke (2000). According to their work, the size and density of the seeds were determined to be 0.58 mm and 225 kg m^−3^, respectively, and the average release height *H*_r_ was set as 0.144 m. However, the wind speed at the reference height was not given directly (Katul *et al.* 2005), and the architecture of the shrub element (e.g. vegetation porosity) was also not clearly described (Bullock and Clarke 2000). Here, a long-time-averaged wind friction speed of u_*a_ = 0.10 m s^−1^ (corresponding to the speed of 3.25 m s^−1^ at height *H* = 10 m) (following a Weibull distribution law) and a vegetation porosity of θ = 0.3 were assumed. Because the total number of seeds released was not clear, the relative proportion of the seed gathering was used. The relative proportion at a gathering location is the number of seeds at gathering location versus the total number of seeds at all of the gathering locations. The simulated results for the settings described above and the observed data are shown in Fig. 2. The simulated results are in good agreement in nearby regions but do not agree well at greater distance (no seeds were collected at the leeward distance > 3 m in the simulated results). This disagreement is likely mainly caused by the uncertainties in the source distribution of the seeds (related to the effective release height) and the wind information details (particularly for high wind speeds). Therefore, the numerical model is capable of capturing the main features of the observed field data and can produce reasonable outcomes.

## Results

### General influences of the local wind reduction on seed dispersal

**Figure 3.**
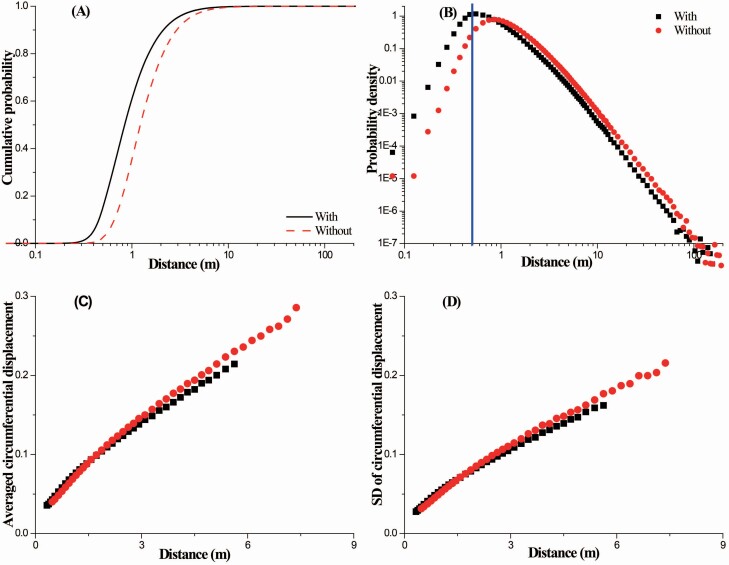
The difference in distributions of wind-dispersed seeds between the cases of considering and not considering local wind reduction in the lee of a single shrub element. ‘With’ or ‘without’ indicates that simulations were conducted with or without considering the effect of the local wind reduction. In (A) solid and dashed lines correspond to ‘with’ and ‘without’, respectively. In (B–D) square and circle symbols correspond to ‘with’ and ‘without’, respectively. In (B) blue solid line indicates the division between the vegetation zone and the leeward wind reduction zone. *H* = 0.5 m, *H*_r_ = 0.5*H*, θ = 0.5 and u_*a_ = 0.30 m s^−1^.

Based on the comparison of the distribution pattern of the deposited seeds that considers the effect of the local wind reduction zone and the distribution pattern that does not consider the effect of the local wind reduction zone, the local wind reduction does affect the dispersal of seeds (Fig. 3). For the figure, the average friction speed (u_*a_) is 0.30 m s^−1^, the release height (*H*_r_) is half the shrub height and the shrub porosity θ is 0.5. The L50 (0.85 m) obtained for the case in which the local wind reduction is considered is reduced by 32 % lower than that (1.25 m) obtained for the case in which the local wind reduction is not considered. Similarly, the L99 is 29 % (5.625 m) lower when the local wind reduction is considered than when it is not considered (7.375 m). The results show that the inclusion of the local wind reduction does not change the distribution pattern of the deposited seeds. However, it does increase the probability of seeds being deposited in the nearby region, and thus, it decreases the probability of seeds being deposited in regions farther away. Moreover, the average circumferential displacement $\bar{dc}$ (Fig. 3C) and the SD of the circumferential displacement $\sigma_{dc}$ (Fig. 3D) linearly increase as the streamwise distance increases both with and without considering the effect of the local wind reduction. However, in comparison to the case in which the local wind reduction is considered, seeds are dispersed farther away in the case in which the local wind reduction zone is not considered, which suggests that they could be dispersed over a wider area. Based on the comparison of the case that considers the local wind reduction and the case that does not consider it, the simulated data reveal that an ~25 % decrease occurs at the maximum $\bar{dc}$ (0.21 m vs. 0.28 m), and an ~27 % decrease occurs at the maximum $\sigma_{dc}$ (0.16 m vs. 0.22 m) under the current wind intensity. These results suggest that not considering the local wind reduction could lead to the overestimation of seed dispersal in the circumferential direction.

### The impacts of wind intensity

As expected, increasing the wind speed led to an increase in the probability that seeds were deposited at distances farther from the vegetation element **[see****Supporting Information—Fig. S3A****and****B****]**, both with and without considering local wind reduction. Here, a uniform wind intensity (represented by u_*_) was used rather than natural wind conditions. The change in the wind speed did not alter the linear responses of either $\bar{dc}$ or $\sigma_{dc}$ to the streamwise distance **[see****Supporting Information—Fig. S3C****and****D****]**. The collected characteristic distances (L50 and L99) are presented in Table 2. The maximum values of both $\bar{dc}$ and $\sigma_{dc}$, which were captured at the location where the cumulative probability reached 99 %, are presented in Table 3. The L50, L99, maximum $\bar{dc}$ and maximum $\sigma_{dc}$ all monotonously increased with increasing wind intensity. The variations in L50, L99, the maximum $\bar{dc}$ and the maximum $\sigma_{dc}$ with increasing wind intensity were fitted using the least square method (in the software Origin 8.0; May and Stevenson 2009).

**Table 2.** Variations of the characteristic distances (L50 and L99) in seed dispersal from a single shrub element with wind intensity (represented by u_*_). The table headings ‘with’ or ‘without’ indicates that simulations were conducted with or without considering the effect of the local wind reduction. *H* = 0.5 m, *H*_r_ = 0.5H and θ = 0.5.

u_*_ (m s^−1^)	L50 (m)		L99 (m)	
	With	Without	With	Without
0.2	0.475	0.650	1.00	1.350
0.3	0.750	1.050	2.175	2.775
0.4	1.050	1.575	4.200	5.25
0.5	1.500	2.025	7. 50	9.50
0.6	2.025	2.625	13.25	16.250

**Table 3.** Variations of the maximum circumferential displacement ($\bar{dc}$) and the SD of the circumferential displacement ($\sigma_{dc}$) in seed dispersal with wind intensity (u_*_). ‘With’ or ‘without’ indicates that simulations were conducted with or without considering the effect of the local wind reduction. *H* = 0.5 m, *H*_r_ = 0.5H and θ = 0.5.

u_*_ (m s^−1^)	Max $\bar{dc}$ (m)		Max $\sigma_{dc}$ (m)	
	With	Without	With	Without
0.2	0.054	0.065	0.042	0.050
0.3	0.102	0.125	0.078	0.096
0.4	0.169	0.210	0.129	0.161
0.5	0.266	0.326	0.203	0.250
0.6	0.406	0.501	0.315	0.382

The results indicate that L50, L99, the maximum $\bar{dc}$ and the maximum $\sigma_{dc}$ increase exponentially with increasing wind intensity (*R*^2^ > 0.99 for all cases), both with and without considering the local wind reduction. The curve for L50 is $L50=A_{50}[\exp(k_{50}u_{*})-1]$. The fitting constants are *A*_50,w_ = 0.92 and *k*_50,w_ = 1.94 for the case in which the local wind reduction is considered (represented by the subscript ‘w’), and the fitting constants are *A*_50,nw_ = 2.45 and *k*_50,nw_ = 1.21 for the case in which the local wind reduction is not considered (represented by the subscript ‘nw’). The curve for L99 is $L99=A_{99}[\exp(k_{99}u_{*})-1]$. The fitting constants are *A*_99,w_ = 0.57, *k*_99,w_ = 5.31, *A*_99,nw_ = 0.80 and *k*_99,nw_ = 5.10. The curve for the maximum $\bar{dc}$ is $\bar{dc}=A_{dc}[\exp(k_{dc}u_{*})-1]$. The fitting constants are $A_{dc,w}$ = 0.05, $k_{dc,w}$ = 3.69, *A*_dc,nw_ = 0.06 and *k*_dc,nw_ = 3.73. The curve for the maximum $\sigma_{dc}$ is $\sigma_{dc}=A_{\sigma }[\exp(k_{\sigma }u_{*})-1]$. The fitting constants are $A_{\sigma ,w}$ = 0.04, $k_{\sigma ,w}$ = 3.63, $A_{\sigma ,nw}$ = 0.05 and $k_{\sigma ,nw}$ = 3.59. The rates of increase (i.e. *k*-values) for L99, the maximum $\bar{dc}$ and the maximum $\sigma_{dc}$ for the case in which the local wind reduction is considered are slightly different than those for the case in which the local wind reduction is not considered. However, the rate of increase of L50 for the case in which the local wind reduction is considered is ~1.6 times that for the case in which the local wind reduction is not considered. Based on the fitting curves described above, the ratio (RL50) of the L50 considering the local wind reduction versus the L50 not considering it gradually increases from 0.64 to 0.76 as the friction speed u_*_ increases from 0.2 to 0.6 m s^−1^.

### The impacts of the release height

The simulation data reveal that decreasing the release height resulted in more seeds being deposited in the nearby region both with and without considering local wind reduction **[see****Supporting Information—Fig. S4A****and****B****]**. The change in the release height did not alter the linear responses of either $\bar{dc}$ or $\sigma_{dc}$ to the streamwise distance **[see****Supporting Information—Fig. S4C****and****D****]**. The characteristic distances (L50 and L99) are presented in Table 4, and the maximum $\bar{dc}$ and the maximum $\sigma_{dc}$ are presented in Table 5.

**Table 4.** Variation of the characteristic distances (L50 and L99) in seed dispersal with release height (*H*_r_). ‘With’ or ‘without’ indicates that simulations were conducted with or without considering the effect of the local wind reduction. *H* = 0.5 m, u_*_ = 0.5 m s^−1^ and θ = 0.5.

*H*_r_/*H*	L50 (m)		L99 (m)	
	With	Without	With	Without
1.0	4.200	4.300	19.750	19.750
0.9	3.700	3.900	17.000	17.875
0.7	2.550	2.925	12.250	13.250
0.5	1.500	2.025	7.500	9.500
0.3	0.650	1.200	3.500	5.625

**Table 5.** Variation in the maximum circumferential displacement ($\bar{dc}$) and the SD of the circumferential displacement ($\sigma_{dc}$) in seed dispersal with release height (*H*_r_). ‘With’ or ‘without’ indicates that simulations were conducted with or without considering the effect of the local wind reduction. *H* = 0.5 m, u_*_ = 0.5 m s^−1^ and θ = 0.5.

*H*_r_/*H*	Max $\bar{dc}$ (m)		Max $\sigma_{dc}$ (m)	
	With	Without	With	Without
1.0	0.648	0.643	0.498	0.489
0.9	0.538	0.586	0.410	0.445
0.7	0.423	0.434	0.314	0.335
0.5	0.266	0.326	0.203	0.250
0.3	0.140	0.213	0.108	0.162

It was determined that L50, L99, the maximum $\bar{dc}$ and the maximum $\sigma_{dc}$ increase with increasing release height. Further curve fittings suggest that they all linearly increase with release height. The curve for L50 is $L50=B_{50}+l_{50}H_{0}/H$; and the fitting constants are *B*_50,w_ = −0.99, *l*_50,w_ = 5.16, *B*_50,nw_ = −0.18 and *l*_50,nw_ = 4.49. The curve for L99 is $L99=B_{99}+l_{99}H_{0}/H$; and the fitting constants are *B*_99,w_ = −3.81, *l*_99,w_ = 23.25, *B*_99,nw_ = −0.63 and *l*_99,nw_ = 20.34. The curve for the maximum $\bar{dc}$ is $\bar{dc}=B_{dc}+l_{dc}H_{0}/H$; and the fitting constants are $B_{dc,w}$ = −0.08, $l_{dc,w}$ = 0.71, *B*_dc,nw_ = 0.02 and *l*_dc,nw_ = 0.62. The curve for the maximum $\sigma_{dc}$ is $\sigma_{dc}=B_{\sigma }+l_{\sigma }H_{0}/H$; and the fitting constants are $B_{\sigma ,w}$ = −0.06, $l_{\sigma ,w}$ = 0.54, $B_{\sigma ,nw}$ = −0.02 and $l_{\sigma ,nw}$ = 0.47. Similar to wind intensity, L50 exhibits the highest sensitivity to the change in the release height (referring to the values of *l*) compared to the other three quantities (i.e. L99, the maximum $\bar{dc}$ and the maximum $\sigma_{dc}$).

### The impacts of the shrub porosity

**Figure 4.**
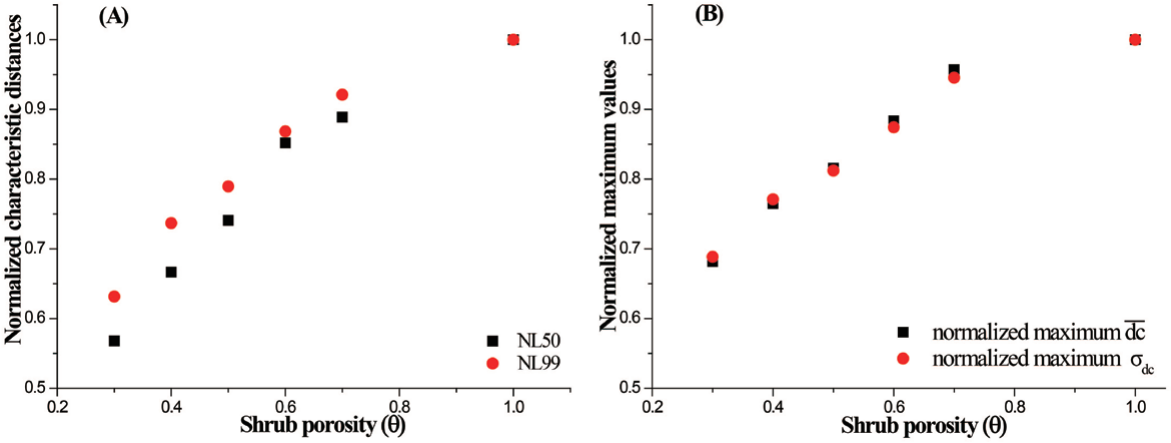
The normalized characteristic distances NL50 and NL99 (A); and the normalized maximum circumferential displacement ($\bar{dc}$) and the SD of the circumferential displacement ($\sigma_{dc}$) (B) versus shrub porosity (θ). *H* = 0.5 m, *H*_r_ = 0.5*H* and u_*_ = 0.5 m s^−1^.

Generally, the normalized L50 (NL50), normalized L99 (NL99), normalized maximum $\bar{dc}$ and normalized maximum $\sigma_{dc}$ all increase as the shrub porosity increases (Fig. 4), which suggests that more seeds are deposited at farther distances from the vegetation element, and the dispersal in the circumferential direction is enhanced. The details of the variations in the cumulative probability, probability density, average circumferential displacement and SD of the circumferential displacement with the streamwise distance are shown in **Supporting Information—Fig. S5**. Additionally, the NL50s were smaller than the NL99s before the shrub porosity reached 1.0 (Fig. 4A), which also implies that the local wind reduction has a greater effect on short-distance dispersal than on long-distance dispersal.

The curve fittings suggest that the cumulative probabilities of the different shrub porosities could be expressed as a logistic function (*R*^2^ > 0.98; **see****Supporting Information—Fig. S6**), that is, Equation (6):


$$\begin{array}{l} y=A_{2}+(A_{1}-A_{2})/[1+{\left(x/x_{0}\right)}^{\;p}] \end{array}$$ (6)

where the initial value is *A*_1_ = 0; the final value is *A*_2_ = 1.0; *x*_0_ is the location at which the cumulative probability reaches 50 % (the same as the L50 defined above); and *p* is the power that describes the shape variation of the fitting curve.

**Figure 5.**
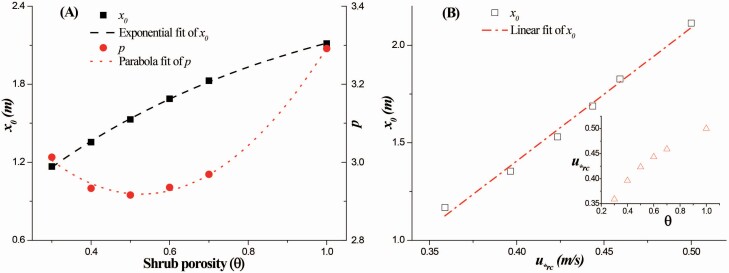
The parameters *x*_0_ and *p* versus shrub porosity (θ) (A) and the parameter *x*_0_ versus the distance-weighted wind intensity (u_*rc_) (B). *H* = 0.5 m, *H*_r_ = 0.5*H* and u_*_ = 0.5 m s^−1^.

The key fitting parameters, *x*_0_ and *p*, of the different shrub porosities are presented in Table 6. The fitting of *x*_0_ is in excellent agreement with the collected L50 from the simulation data **[see****Supporting Information—Fig. S7****]**. The power values of *p* decrease slightly at first and then increase as the shrub porosity increases, reflecting the main variation of the shape of the cumulative probability curve. The data analysis (Fig. 5A) reveals that *x*_0_ increases exponentially as the shrub porosity increases: *x*_0_ = 2.69 − 2.33exp(−1.41θ) (*R*^2^ > 0.99). In addition, *p* changes with shrub porosity in the form of a parabola: *p* = 3.38 − 1.77θ + 1.68θ^2^ (*R*^2^ > 0.99). Furthermore, a distance-weighted streamwise wind intensity, $u_{*rc}=\int_{0}^{L_{x}}u_{*s}(x,0,0)dx/L_{x}$, is proposed for the local wind reduction region. The variation in u_*rc_ with shrub porosity (insert in Fig. 5B) is similar to that of *x*_0_ (Fig. 5A). However, *x*_0_ increases linearly as the shrub porosity increases: *x*_0_ = 6.84u_*rc_ − 1.33 (*R*^2^ > 0.98). This suggests that the effect of shrub porosity on short-distance dispersal may be reflected by its role in altering the distance-weighted wind intensity within local wind reduction region.

**Table 6.** Variations of key fitting parameters *x*_0_ and *p* as well the corresponding standard errors with shrub porosity (θ). *H* = 0.5 m, *H*_r_ = 0.5*H* and u_*_ = 0.5 m s^−1^. *x*_0_: the location at which the cumulative probability reaches 50 %, and *p*: the power index that describes the shape variation of the fitting curve.

θ	1.0	0.7	0.6	0.5	0.4	0.3
*x*_0_ (m)	2.114	1.827	1.688	1.529	1.354	1.167
SE of *x*_0_	0.0080	0.0065	0.0062	0.0060	0.0057	0.0052
*p*	3.292	2.969	2.936	2.916	2.933	3.013
SE of *p*	0.0356	0.0271	0.0274	0.0289	0.0317	0.0352

## Discussion

In this study, the wind dispersal of seeds released from a point source above an open landscape that is sparsely populated with shrubs was simulated by parameterizing the spatial distribution of the wind speed around a single shrub element (mainly considering the local wind reduction both within and in the lee of the shrub element) based on previous field observations (Leenders *et al.* 2011; Mayaud *et al.* 2017). The main findings obtained from the simulation are discussed below. These results increase our understanding of the reasons for the disagreement between theoretical studies and field observations and help to determine possible directions for future studies.

Although the effect of wind reduction within forests on seed dispersal has been studied (Nathan *et al.* 2002b, 2011), local wind reduction has not been considered when modelling the dispersal of seeds in an open landscape (Bullock and Clarke 2000; Nathan *et al.* 2002a), for example, seed dispersal from a shrub element in a sparsely vegetated area. However, the simulated results obtained in this study reveal that local wind reduction has a significant effect on seed dispersal in the streamwise and circumferential directions (Fig. 3), which suggests that the effect of local wind reduction should not be ignored. It should be noted that the size of the wind reduction zone is finite. Thus, the effect of local wind reduction on seed dispersal is limited, particularly for seeds that could quickly escape the wind reduction zone and have a high potential for long-distance dispersal. Therefore, local wind reduction is likely to exert a greater impact on short-distance dispersal than on long-distance dispersal.

These finding may be crucial for predicting shrub succession, which plays an important role in hindering erosion and reducing sediment transport (Leenders *et al.* 2007; Okin 2008; Leenders *et al.* 2011) in degraded and desert areas. In these areas, shrub reproduction is likely to depend on the amount of seeds in the soil seed bank because the germination of seeds and the survival of seedlings are difficult because of the extremely poor conditions in these environments (Clauss and Venable 2000). Therefore, short-distance dispersal (or seed deposition near the source region), which usually has a high probability density, is more ecologically meaningful for variations in local vegetation patterns than long-distance dispersal, which has a very low probability density, in these areas (Venable *et al.* 2008). However, the effect of local wind reduction on long-distance dispersal should not be overlooked.

In areas where suitable seed germination conditions exist, the long-distance dispersal of seeds is still crucial for the expansion and invasion of vegetation (Jongejans *et al.* 2008). A small difference in predicting the probability of long-distance dispersal may lead to a large mistake in the estimation of the change of species over time, such as in the case of responses to global climate change (Bullock *et al.* 2012; Corlett and Westcott 2013). Thus, previous studies that did not consider local wind reduction in open landscape cases (Bullock and Clarke 2000; Nathan *et al.* 2002a) may have underestimated the deposition of seeds in nearby regions and thereby have overestimated the probability of long-distance dispersal.

However, the effect of local wind reduction on short-distance dispersal weakens as the wind intensity and release height increase (Tables 2–5). The height of shrub vegetation is typically <10 m (Bullock and Clarke 2000; Mayaud *et al.* 2017), and the wind speed below this height is usually not very high (referring to Equation (1)), except during extreme weather events. Thus, the effect of wind intensity should be considered. Although we knew that seeds are spatially distributed in shrub vegetation according to the vegetation’s structure (Cousens and Rawlinson 2001), when modelling or predicting the kernels of the dispersed seeds, previous studies have generally assumed that the seeds are released from an average or equivalent height (Bullock and Clarke 2000; Nathan *et al.* 2002a, b). The effect of the release height determined in this study (Tables 2 and 3) indicates that such treatment may lead to a greater difference between a modelled kernel and a real dispersed kernel.

The seeds distributed in the upper part of the vegetation or in a windward area would have the advantage of encountering a high wind speed. Thus, the effect of local wind reduction on their dispersal would be reduced to some extent. Additionally, the seeds distributed in a windward area may need to bypass the foliage canopy, which can also reduce their dispersal. The seeds distributed in lower locations or in a leeward area would encounter a lower wind speed and thus be significantly affected by local wind reduction. It has been observed that a difference in wind speed (upper vs. lower locations and windward vs. leeward areas) could lead to temporally and spatially heterogeneous seed release (Greene and Johnson 1995). This increases the difficulty of accurately predicting the dispersal kernels based on available meteorological data.

Typically, seeds are dispersed both wider and farther away as the shrub porosity increases (Figs 4 and 5). Changes in shrub porosity could remarkably alter the dispersal kernels under a given wind intensity. Because of the diversity of vegetation structures, the vegetation porosity differs among species, and it could also vary with time due to seasonal and growth stage changes. Therefore, field observed dispersal kernels in different locations could be diverse even if the wind intensities are almost identical (Nathan *et al.* 2000). Consequently, empirical fitting curves cannot be applied to other dispersal conditions (Bullock *et al.* 2017). Thus, it is worth including porosity into the distribution function of dispersed seeds.

The simulated results reveal that the cumulative probability of the dispersed seeds along the streamwise direction could be expressed well by a logistic function **[see****Supporting Information—Fig. S6A****]**. There are two main reasons why the commonly used log-normal probability density function was not employed in this study. First, the characteristic distance L50 is more optimally predicted using a logistic function as opposed to a log-normal probability density function **[see****Supporting Information—Figs S6****and****S7****]**. The curve fitting using a log-normal function was less accurate in terms of the predictions in both the streamwise location and the value of the peak probability density **[see****Supporting Information—Fig. S6C****and****D****]**. Second, compared to the probability density function, the cumulative probability function is only streamwise distance-dependent and is independent of the size of the collecting grids.

The distance-weighted wind intensity, u_*rc_, in which shrub porosity is included, was introduced to describe the *x*_0_ where the cumulative probability reaches 50 % (Fig. 5B). The success of this introduction should be based on the fact that the majority of seeds were always deposited within the wind reduction zone under current simulation settings. However, such an attempt is still empirical, and the theoretical derivation based on physical and mathematical rules may obtain not only quantitative but also general results. Although the simulated data under the current specific settings support the selection of a logistic function, the logistic function sometimes performs poorly in locations where the cumulative probability is <10 %. Disagreement between the fitting curves and the original data is a common problem in data analyses (both field and simulation data) (Bullock *et al.* 2017, 2018; Cousens *et al.* 2018). Therefore, it would be beneficial to find a reliable method, e.g. a density function, cumulative function (Brown and Mayer 1988; Schippers and Jongejans 2005) or survival function (McNair *et al.* 2012) to describe the dispersal kernels in the field or for simulation data analysis of long-distance dispersal.

In this study, all of the above results and discussion mentioned above are for a single shrub element, and they were obtained by employing the spatial distribution of the wind speed constructed based on existing measurements (Mayaud *et al.* 2017). These measurements may capture the main features of the average wind speed around the shrub element, but the designed spatial grids used for the measurement are too large to confirm the exact shape or the boundary of the wind reduction region. There is also a lack of measurements of the wind variation within the shrub element (Mayaud *et al.* 2017). The height of the vegetation is another important factor affecting the local wind reduction region (Okin 2008; Leenders *et al.* 2011; Mayaud *et al.* 2017). Its effects on the description of the local wind reduction and seed dispersal require further investigation. Generally, the description of the wind speed around a single shrub element is empirical and simple. Additional studies would be valuable if they considered both the vertical and lateral wind speeds within the local wind reduction region. This requires not only studies in which ideally rigid vegetation models (both solid and porous) (e.g. Krajnović 2011; Rostamy *et al.* 2012; Lv *et al.* 2014; Taddei *et al.* 2016; Fu *et al.* 2019) are used, but also studies in which real and flexible vegetation (e.g. Walter *et al.* 2012; Miri *et al.* 2017; Kang *et al.* 2019) are used.

The shape changes and dynamic responses of vegetation to the incoming air flow may alter the vertical and lateral wind speeds and thus affects seed dispersal. Additionally, the effect of local wind reduction in cases with multiple vegetation elements is not yet clear. Large eddy simulation may be a good choice (Bohrer *et al.* 2008; Dupont and Brunet 2008; Pan *et al.* 2015) for dense vegetation cases; however, the huge computational costs arising from the grid precision, computation domain, and coupling dynamics of the air flow and vegetation are still obstacles that prevent us from obtaining reliable wind speed and turbulence values around vegetation, accurate vegetation dynamics data and subsequently, accurate information regarding the dispersal of seeds.

Finally, the effect of local wind reduction is not limited to shrub vegetation, and it may also apply to other low vegetation. For instance, it may be conditionally suitable for low trees with large crowns in an open landscape. However, the basic physical model of a tree is typically different from that of a shrub (Liu *et al.* 2018). Studies of the parameterization of the wind reduction region in the lee of a tree should initially focus on the differences in the geometric shape and height of the tree compared to that of a shrub, followed by the subsequent application of this method to other vegetation types.

## Supporting Information

The following additional information is available in the online version of this article—

**Figure S1.** The distribution of surface friction velocity (u_*s_) along streamwise direction.

**Figure S2.** The definition of the circumferential displacement (dc) in circumferential direction.

**Figure S3.** The variation of the effect of local wind reduction on seed dispersal with the change of wind intensity.

**Figure S4.** The variation of the effect of local wind reduction on seed dispersal with the change of release height.

**Figure S5.** The distributions of deposited seeds with the change of the porosity of a single shrub element.

**Figure S6.** Curve fittings for cumulative probability and probability density of deposited seeds with the change of the porosity of a single shrub element.

**Figure S7.** Comparison of *x*_0_ obtained by fitting curves with L50 obtained by original simulated data. 

**Raw data excel file.** Raw data in Figs 2–5.

plab025_suppl_Supplementary_Materials

## Data Availability

The supplementary materials and raw data are available as **Supporting Information**.

## Notes

**Populations & Communities. Chief Editor:** Jean Burns

## References

[CIT0001] Anderson RS . 1987. Eolian sediment transport as a stochastic process: the effects of a fluctuating wind on particle trajectories. Journal of Geology 95:497–512.

[CIT0002] Arritt RW , Clark CA, Goggi AS, Sanchez HL, Westgate ME, Riese JM. 2007. Lagrangian numerical simulations of canopy air flow effects on maize pollen dispersal. Field Crops Research 102:151–162.

[CIT0003] Beckman NG , Aslan CE, Rogers HS. 2020a. Introduction to the special issue: the role of seed dispersal in plant populations: perspectives and advances in a changing world. AoB PLANTS 12:plaa010; doi:10.1093/aobpla/plaa010 32337017 PMC7164217

[CIT0004] Beckman NG , Aslan CE, Rogers HS, Kogan O, Bronstein JL, Bullock JM, Hartig F, HilleRisLambers J, Zhou Y, Zurell D, Brodie JF, Bruna EM, Cantrell RS, Decker RR, Efiom E, Fricke EC, Gurski K, Hastings A, Johnson JS, Loiselle BA, Miriti MN, Neubert MG, Pejchar L, Poulsen JR, Pufal G, Razafindratsima OH, Sandor ME, Shea K, Schreiber S, Schupp EW, Snell RS, Strickland C, Zambrano J. 2020b. Advancing an interdisciplinary framework to study seed dispersal ecology. AoB PLANTS 12:plz048; doi:10.1093/aobpla/plz048 32346468 PMC7179845

[CIT0005] Bohrer G , Katul GG, Nathan R, Walko RL, Avissar R. 2008. Effects of canopy heterogeneity, seed abscission and inertia on wind-driven dispersal kernels of tree seeds. Journal of Ecology 96:569–580.

[CIT0006] Brown RF , Mayer DG. 1988. Representing cumulative germination. 2. The use of the Weibull function and other empirically derived curves. Annals of Botany 61:127–138.

[CIT0007] Bullock JM , Clarke RT. 2000. Long distance seed dispersal by wind: measuring and modelling the tail of the curve. Oecologia 124:506–521. 28308389 10.1007/PL00008876

[CIT0008] Bullock JM , Hooftman DA, Tamme R, Götzenberger L, Pärtel M, Mallada González L, White SM. 2018. All dispersal functions are wrong, but many are useful: a response to Cousens *et al*. Journal of Ecology 106:907–910.

[CIT0009] Bullock JM , Mallada González L, Tamme R, Götzenberger L, White SM, Pärtel M, Hooftman DA. 2017. A synthesis of empirical plant dispersal kernels. Journal of Ecology 105:6–19.

[CIT0010] Bullock JM , White SM, Prudhomme C, Tansey C, Perea R, Hooftman DA. 2012. Modelling spread of British wind-dispersed plants under future wind speeds in a changing climate. Journal of Ecology 100:104–115.

[CIT0011] Chen Z , Ortiz A, Zong L, Nepf H. 2012. The wake structure behind a porous obstruction and its implications for deposition near a finite patch of emergent vegetation. Water Resources Research 48:W09517.

[CIT0012] Cheng NS . 1997. Simplified settling velocity formula for sediment particle. Journal of Hydraulic Engineering 123:149–152.

[CIT0013] Clauss MJ , Venable DL. 2000. Seed Germination in desert annuals: an empirical test of adaptive bet hedging. The American Naturalist 155:168–186. 10.1086/303314 10686159

[CIT0014] Corlett RT , Westcott DA. 2013. Will plant movements keep up with climate change? Trends in Ecology & Evolution 28:482–488. 23721732 10.1016/j.tree.2013.04.003

[CIT0015] Cousens RD , Hughes BD, Mesgaran MB. 2018. Why we do not expect dispersal probability density functions based on a single mechanism to fit real seed shadows. Journal of Ecology 106:903–906.

[CIT0016] Cousens RD , Rawlinson AA. 2001. When will plant morphology affect the shape of a seed dispersal “kernel”? Journal of Theoretical Biology 211:229–238. 11444954 10.1006/jtbi.2001.2341

[CIT0017] Csanady GT . 1963. Turbulent diffusion of heavy particles in the atmosphere. Journal of the Atmospheric Sciences 20:201–208.

[CIT0018] Dorp DV , Hoek WVD, Daleboudt C. 1996. Seed dispersal capacity of six perennial grassland species measured in a wind tunnel at varying wind speed and height. Canadian Journal of Botany 74:1956–1963.

[CIT0019] Duman T , Trakhtenbrot A, Poggi D, Cassiani M, Katul GG. 2016. Dissipation intermittency increases long-distance dispersal of heavy particles in the canopy sublayer. Boundary-Layer Meteorology 159:41–68.

[CIT0020] Dupont S , Brunet Y. 2008. Edge flow and canopy structure: a large-eddy simulation study. Boundary-Layer Meteorology 126:51–71.

[CIT0021] Fu LT , Fan Q, Huang ZL. 2019. Wind speed acceleration around a single low solid roughness in atmospheric boundary layer. Scientific Reports 9:1–11. 31427684 10.1038/s41598-019-48574-7 PMC6700104

[CIT0022] Greene DF , Johnson EA. 1989. A model of wind dispersal of winged or plumed seeds. Ecology 70:339–347.

[CIT0023] Greene DF , Johnson EA. 1995. Long-distance wind dispersal of tree seeds. Canadian Journal of Botany 73:1036–1045.

[CIT0024] Greene DF , Johnson EA. 1996. Wind dispersal of seeds from a forest into a clearing. Ecology 77:595–609.

[CIT0025] Greene DF , Quesada M. 2005. Seed size, dispersal, and aerodynamic constraints within the Bombacaceae. American Journal of Botany 92:998–1005. 21652484 10.3732/ajb.92.6.998

[CIT0026] Heydel F , Cunze S, Bernhardt-Römermann M, Tackenberg O. 2014. Long-distance seed dispersal by wind: disentangling the effects of species traits, vegetation types, vertical turbulence and wind speed. Ecological Research 29:641–651.

[CIT0027] Hildebrand FB . 1956. Introduction to numerical analysis. New York: McGraw-Hill.

[CIT0028] Howe HF , Smallwood J. 1982. Ecology of seed dispersal. Annual Review of Ecology Evolution and Systematics 13:201–228.

[CIT0029] Jarosz N , Loubet B, Huber L. 2004. Modelling airborne concentration and deposition rate of maize pollen. Atmospheric Environment 38:5555–5566.

[CIT0030] Jongejans E , Skarpaas O, Shea K. 2008. Dispersal, demography and spatial population models for conservation and control management. Perspectives in Plant Ecology, Evolution and Systematics 9:153–170.

[CIT0031] Kaimal JC , Finnigan JJ. 1994. Atmospheric boundary layer flows: their structure and measurement. New York: Oxford University Press.

[CIT0032] Kang L , Zhang J, Zou X, Cheng H, Zhang C, Yang Z. 2019. Experimental investigation of the aerodynamic roughness length for flexible plants. Boundary-Layer Meteorology 172:1–20.

[CIT0033] Katul GG , Porporato A, Nathan R, Siqueira M, Soons MB, Poggi D, Horn HS, Levin SA. 2005. Mechanistic analytical models for long-distance seed dispersal by wind. The American Naturalist 166:368–381. 10.1086/432589 16224691

[CIT0034] Kawamura T , Hiwada M, Hibino T, Mabuchi I, Kumada M. 1984. Flow around a finite circular cylinder on a flat plate: cylinder height greater than turbulent boundary layer thickness. Bulletin of JSME 27:2142–2151.

[CIT0035] Kok JF , Renno NO. 2009. A comprehensive numerical model of steady state saltation (COMSALT). Journal of Geophysical Research: Atmosphere 114:D17204.

[CIT0036] Krajnović S . 2011. Flow around a tall finite cylinder explored by large eddy simulation. Journal of Fluid Mechanics 676:294–317.

[CIT0037] Kuparinen A , Markkanen T, Riikonen H, Vesala T. 2007. Modeling air-mediated dispersal of spores, pollen and seeds in forested areas. Ecological Modelling 208:177–188.

[CIT0038] Leenders JK , Boxel JV, Sterk G. 2007. The effect of single vegetation elements on wind speed and sediment transport in the Sahelian zone of Burkina Faso. Earth Surface Processes and Landforms 32:1454–1474.

[CIT0039] Leenders JK , Sterk G, Van Boxel JH. 2011. Modelling wind-blown sediment transport around single vegetation elements. Earth Surface Processes and Landforms 36:1218–1229.

[CIT0040] Liu C , Zheng Z, Cheng H, Zou X. 2018. Airflow around single and multiple plants. Agricultural and Forest Meteorology 252:27–38.

[CIT0041] Lv P , Dong Z, Mu Q, Luo W, Qian G. 2014. An analysis of drag force on wind of shrubs simulated in a wind tunnel. Environmental Earth Sciences 71:125–131.

[CIT0042] Maxey MR , Riley JJ. 1983. Equation of motion for a small rigid sphere in a nonuniform flow. Physics of Fluids 26:883–889.

[CIT0043] May RA , Stevenson KJ. 2009. Software review of Origin 8. Journal of the American Chemical Society 131:872–872.

[CIT0044] Mayaud J , Webb N. 2017. Vegetation in drylands: effects on wind flow and aeolian sediment transport. Land 6:64.

[CIT0045] Mayaud JR , Wiggs GF, Bailey RM. 2017. A field-based parameterization of wind flow recovery in the lee of dryland plants. Earth Surface Processes and Landforms 42:378–386.

[CIT0046] McNair JN , Sunkara A, Frobish D. 2012. How to analyse seed germination data using statistical time-to-event analysis: non-parametric and semi-parametric methods. Seed Science Research 22:77–95.

[CIT0047] Miri A , Dragovich D, Dong Z. 2017. Vegetation morphologic and aerodynamic characteristics reduce aeolian erosion. Scientific Reports 7:12831. 28993700 10.1038/s41598-017-13084-x PMC5634502

[CIT0048] Nathan R , Horn HS, Chave J, Levin SA. 2002a. Mechanistic models for tree seed dispersal by wind in dense forests and open landscapes. In: Levey DJ, Silva WR, Galetti M, eds. Seed dispersal and frugivory: ecology, evolution and conservation. Wallingford, UK: CAB International, 69–82.

[CIT0049] Nathan R , Katul GG, Bohrer G, Kuparinen A, Soons MB, Thompson SE, Trakhtenbrot A, Horn HS. 2011. Mechanistic models of seed dispersal by wind. Theoretical Ecology 4:113–132.

[CIT0050] Nathan R , Katul GG, Horn HS, Thomas SM, Oren R, Avissar R, Pacala SW, Levin SA. 2002b. Mechanisms of long-distance dispersal of seeds by wind. Nature 418:409–419. 12140556 10.1038/nature00844

[CIT0051] Nathan R , Safriel UN, Noy-Meir I, Schiller G. 2000. Spatiotemporal variation in seed dispersal and recruitment near and far from *Pinus halepensis* trees. Ecology 81:2156–2169.

[CIT0052] Nathan R , Safriel UN, Noy-Meir I. 2001. Field validation and sensitivity analysis of a mechanistic model for tree seed dispersal by wind. Ecology 82:374–388.

[CIT0053] Nishimura K , Hunt JCR. 2000. Saltation and incipient suspension above a flat particle bed below a turbulent boundary layer. Journal of Fluid Mechanics 417:77–102.

[CIT0054] Okin GS . 2008. A new model of wind erosion in the presence of vegetation. Journal of Geophysical Research: Earth Surface 113:F02S10.

[CIT0055] Pan Y , Chamecki M, Isard SA, Nepf HM. 2015. Dispersion of particles released at the leading edge of a crop canopy. Agricultural and Forest Meteorology 211:37–47.

[CIT0056] Raupach M . 1992. Drag and drag partition on rough surfaces. Boundary-Layer Meteorology 60:375–395.

[CIT0057] Raupach MR , Antonia RA, Rajagopalan S. 1991. Rough-wall turbulent boundary layers. Applied Mechanics Reviews 44:1–25.

[CIT0058] Rostamy N , Sumner D, Bergstrom DJ, Bugg JD. 2012. Local flow field of a surface-mounted finite circular cylinder. Journal of Fluids and Structures 34:105–122.

[CIT0059] Sadique J , Yang XI, Meneveau C, Mittal R. 2017. Aerodynamic properties of rough surfaces with high aspect-ratio roughness elements: effect of aspect ratio and arrangements. Boundary-Layer Meteorology 163:203–224.

[CIT0060] Schippers P , Jongejans E. 2005. Release thresholds strongly determine the range of seed dispersal by wind. Ecological Modelling 185:93–103.

[CIT0061] Seguro JV , Lambert TW. 2000. Modern estimation of the parameters of the Weibull wind speed distribution for wind energy analysis. Journal of Wind Engineering and Industrial Aerodynamics 85:75–84.

[CIT0062] Taddei S , Manes C, Ganapathisubramani B. 2016. Characterisation of drag and wake properties of canopy patches immersed in turbulent boundary layers. Journal of Fluid Mechanics 798:27–49.

[CIT0063] Tang T , Yu P, Shan X, Chen H. 2019. The formation mechanism of recirculating wake for steady flow through and around arrays of cylinders. Physics of Fluids 31:043607.

[CIT0064] Thomson DJ . 1987. Criteria for the selection of stochastic models of particle trajectories in turbulent flows. Journal of Fluid Mechanics 180:529–556.

[CIT0065] Trakhtenbrot A , Katul GG, Nathan R. 2014. Mechanistic modeling of seed dispersal by wind over hilly terrain. Ecological Modelling 274:29–40.

[CIT0066] Van Dop H , Nieuwstadt FTM, Hunt JCR. 1985. Random walk models for particle displacements in inhomogeneous unsteady turbulent flows. Physics of Fluids 28:1639–1653.

[CIT0067] Venable DL , Flores-Martinez A, Muller-Landau HC, Barron-Gafford G, Becerra JX. 2008. Seed dispersal of desert annuals. Ecology 89:2218–2227. 18724732 10.1890/07-0386.1

[CIT0068] Walter B , Gromke C, Leonard KC, Manes C, Lehning M. 2012. Spatio-temporal surface shear-stress variability in live plant canopies and cube arrays. Boundary-Layer Meteorology 143:337–356.

[CIT0069] Wilson JD . 2000. Trajectory models for heavy particles in atmospheric turbulence: comparison with observations. Journal of Applied Meteorology 39:1894–1912.

[CIT0070] Wilson JD , Sawford BL. 1996. Review of Lagrangian stochastic models for trajectories in the turbulent atmosphere. Boundary-Layer Meteorology 78:191–210.

[CIT0071] Yang XI , Sadique J, Mittal R, Meneveau C. 2016. Exponential roughness layer and analytical model for turbulent boundary layer flow over rectangular-prism roughness elements. Journal of Fluid Mechanics 789:127–165.

