# Supplementary material for: Effect of the local wind reduction zone on seed dispersal from a single shrub element on sparsely vegetated land
Source: AoB Plants. 2021 May 21;13(4):plab025. doi: 10.1093/aobpla/plab025 (PMC8266638; doi:10.1093/aobpla/plab025)
Supplement: plab025_suppl_Supplementary_Materials [file plab025_suppl_supplementary_materials.zip › plab025_suppl_Supplementary_Materials.docx]

***Supplementary Materials***

**Supplementary Figures**

Figure S1. The distribution of surface friction velocity (u_*s_) along streamwise direction.

Figure S1. The distribution of surface friction velocity (u_*s_) along streamwise direction.

Figure S2. The definition of the circumferential displacement (dc) in circumferential direction.

Figure S2. The definition of the circumferential displacement (dc) in circumferential direction.

Figure S3. The variation of the effect of local wind reduction on seed dispersal with the change of wind intensity.

Figure S3. The variation of the effect of local wind reduction on seed dispersal with the change of wind intensity. H=0.5 m, H_r_=0.5H, and $\theta$=0.5. In panel (A): the solid lines and the dashed lines suggest with and without considering local wind reduction, respectively. In panels (B)-(D): solid scatters and open scatters suggest with and without considering local wind reduction, respectively.

Figure S4. The variation of the effect of local wind reduction on seed dispersal with the change of release height.

Figure S4. The variation of the effect of local wind reduction on seed dispersal with the change of release height. H=0.5 m, u_*_=0.5 m/s, and $\theta$=0.5.

Figure S5. The distributions of deposited seeds with the change of the porosity of a single shrub element.

Figure S5. The distributions of deposited seeds with the change of the porosity of a single shrub element. H=0.5 m, H_r_=0.5H, and u_*_=0.5 m/s.

Figure S6. Curve fittings for cumulative probability and probability density of deposited seeds with the change of the porosity of a single shrub element.

Figure S6. Curve fittings for cumulative probability (Panels (A) and (B)) and probability density (Panels (C) and (D)) of deposited seeds with the change of the porosity of a single shrub element. Dashed lines are fitting curves. Panels (A) and (B): logistic curve fittings, Panels (C) and (D): log-normal curve fittings. H=0.5 m, H_r_=0.5H, and u_*_=0.5 m/s.

Figure S7. Comparison of *x_0_* obtained by fitting curves with L50 obtained by original simulated data

Figure S7. Comparison of *x_0_* obtained by fitting curves with L50 obtained by original simulated data.
